# Generating Realistic and Representative Trajectories with Mobility Behavior Clustering

**DOI:** 10.1145/3589132.3625657

**Published:** 2023-12-22

**Authors:** Haowen Lin, Sina Shaham, Yao-Yi Chiang, Cyrus Shahabi

**Affiliations:** 1 University of Southern California, Department of Computer Science, Los Angeles, United States; 2 University of Minnesota, Department of Computer Science and Engineering, Twin Cities, United States

**Keywords:** trajectory generation, GAIL

## Abstract

Accessing realistic human movements (aka trajectories) is essential for many application domains, such as urban planning, transportation, and public health. However, due to privacy and commercial concerns, real-world trajectories are not readily available, giving rise to an important research area of generating synthetic but realistic trajectories. Inspired by the success of deep neural networks (DNN), data-driven methods learn the underlying human decision-making mechanisms and generate synthetic trajectories by directly fitting real-world data. However, these DNN-based approaches do not exploit people’s moving behaviors (e.g., work commute, shopping purpose), significantly influencing human decisions during the generation process. This paper proposes MBP-GAIL, a novel framework based on generative adversarial imitation learning that synthesizes realistic trajectories that preserve moving behavior patterns in real data. MBP-GAIL models temporal dependencies by Recurrent Neural Networks (RNN) and combines the stochastic constraints from moving behavior patterns and spatial constraints in the learning process. Through comprehensive experiments, we demonstrate that MBP-GAIL outperforms state-of-the-art methods and can better support decision making in trajectory simulations.

## 1 INTRODUCTION

Recent years have witnessed rapid advancements in location-sensing technologies, which enable us to collect a large amount of spatiotemporal trajectory data. While these spatiotemporal data are extremely valuable, only limited sources and small-scale datasets [20] are available to researchers due to privacy restrictions and commercial concerns [4]. Therefore, creating a generator to efficiently synthesize a large number of realistic trajectories is an important research direction.

Traditional rule-based methods assume that predetermined mechanisms can describe individual mobility with a few specific mobility parameters, such as the average time spent staying at each visiting location [5, 10]. However, real-world trajectories exhibit complex transition patterns, which cannot be accurately defined by simple rules [1]. Inspired by the success of deep generative neural networks in computer vision [6, 7, 12], graph [2] and natural language processing[13], there are data-driven generators that leverage real-world data to generate synthetic trajectories. One line of research models human movements as state transitions and formulates trajectory generations as a decision-making process [14]. These approaches apply Generative Adversarial Imitation Learning (GAIL) [3] to generate movements considering individual sequences of actions. [1, 9].

However, existing data-driven trajectory generators are limited in that they assume that the next state is completely decided by mimicking individual human actions, while in the real world, each trajectory typically comes with an underlying purpose that could collectively influence human decisions. For example, knowing that the purpose of our travel is to commute to work suggests that we should start from a residential area and end at a business area (perhaps a stop at a coffee shop on the way). We term such semantic information as the “moving behavior” of trajectories, i.e., the traveling purpose that describes a user’s movement. Lacking the moving behavior information in the trajectory generation process not only limits the applications in developing advanced downstream modeling tasks [18], e.g., precise ads-targeting on locations frequently passed by specific types of moving behaviors, but also makes the generation model less realistic.

This paper proposes a new framework that integrates prior moving behavior patterns into GAIL methods. Incorporating moving behavior is not a trivial task since the raw trajectory coordinates do not contain any useful information indicating the moving behavior. Instead, we generate the “context sequence” for each trajectory from nearby Points-Of-Interest (POIs) and then extend the notion of moving behavior (see Def 1.3) to be defined as the transition patterns of context sequences. Subsequently, we consider the action of a human as a joint decision influenced by past moving histories and context trajectories guided by a specific moving behavior pattern. We jointly incorporate the dynamics of the transitions between raw locations and their context in a generator to learn the movement policy. We also propose a discriminator to differentiate the generated trajectories from the observed real trajectories and a classifier to evaluate moving behavior patterns. Moreover, our framework is flexible to incorporate reasonable inductive bias in trajectory generation, such as the inherent spatial dependencies between the consecutive raw locations. We conduct extensive experiments on real-world data through various evaluation metrics to validate the effectiveness of our approach.

### 1.1 Problem Formulation

#### Definition 1.1 (Mobility Trajectory).

A mobility trajectory is a sequence of spatiotemporal points, i.e., $\tau^{L}=[\tau_{1}^{L},\cdots ,\tau_{N}^{L}]$ where $\tau_{i}^{L}=\left(l_{i},t_{i}\right),\;t_{i}$ is the timestamp, $l_{i}$ is the location, which can be a region identification (ID), and N is the total length of the trajectory.

We preprocess the mobility trajectories and transform the spatiotemporal trajectories into context sequences. For each location, we group and count the POIs located inside its corresponding grid cell, and, without loss of generality, the context type is represented as the category of the most counted POI type, such as Industry, Residential Area, Education, or Health Care. We define a conversion matrix $\Gamma \in {\left\{0,\;1\right\}}^{Q\times K}$ where $\Gamma_{q,k}=1$ if the *k^th^* location belongs to the *q*^*th*^ context type to shows the mapping relation between the locations and the context type.

#### Definition 1.2 (Context Trajectory).

A context trajectory is a chronologically ordered sequence, i.e., $\tau^{C}=[\tau_{1}^{C},\tau_{2}^{C},\cdots ,\tau_{N}^{C}]$ where each element $\tau_{i}^{C}$ is a context-time tuple ($c_{i},t_{i}$), and $c_{i}$ is the location context type, which can be obtained using the mobility trajectory and the conversion matrix by $c_{i}=argmax\left(\Gamma \times OneHot\left(l_{i}\right)\right)$.

#### Definition 1.3 (Moving Behavior).

The moving behavior m∈{1,2,…,M} is the label of grouped trajectories with high proximity to transition patterns in context sequences. We apply DBSCAN clustering [8] to the edit distance [11], a common trajectory distance measurement metric, of the context trajectories to obtain the moving behavior label from the real trajectories.

##### Problem 1 (Realistic and Representative Synthetic Trajectory Generation).

*Given real-world trajectories in a specific area, where each trajectory is associated with a specific moving behavior type* m∈{1,2,…,M}, *the goal is to mimic individuals’ decision-making process and generate synthetic trajectories while retaining the individual and general properties of moving behavior of each trajectory.*

## 2 PROPOSED METHOD OF MBP-GAIL

**Figure 1:**
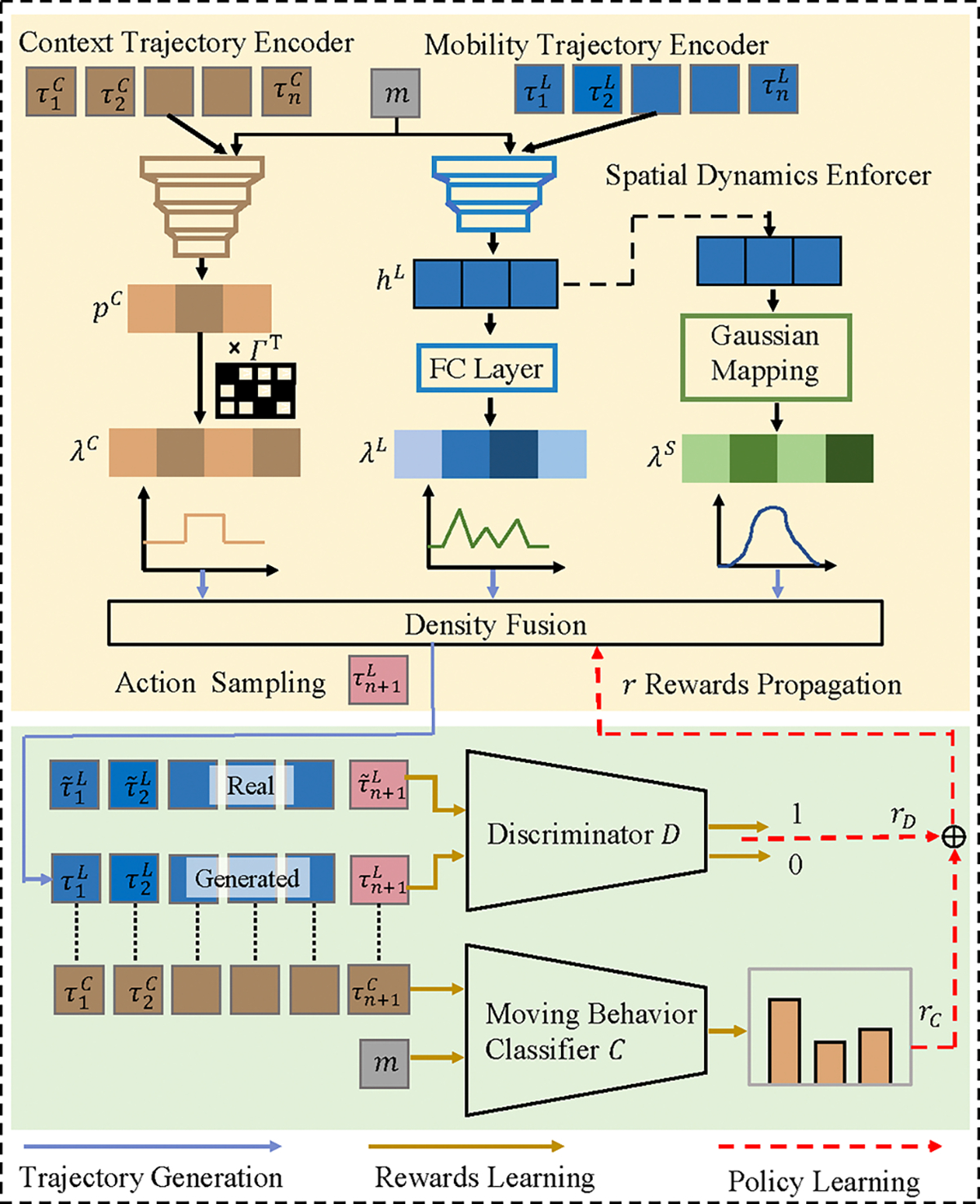
Illustration of the MBP-GAIL framework.

The overview of our MBP-GAIL framework is shown in Fig. 1. MBP-GAIL has two major components. The policy network $\pi_{\theta }$ (the yellow component in Figure 1) as the generator, which, when given the desired moving behavior m, learns to generate an action a similar to the real-world cases based on the current state. The second component, the reward network r (the green component in Figure 1) consists of a discriminator trained to distinguish between policy-generated and real-world cases and a classifier to detect moving behavior patterns. The policy network and the reward function are jointly optimized through the framework of GAIL to solve a minimax problem as follows: [21] :

(1) $$\underset{\psi }{max}\;\underset{\theta }{min}\;ℒ(\theta ,\psi )=E_{(s,a,m)\in {𝒯}_{E}}log\;{𝒟}_{\psi }(s,a,m)+\;E_{(s,a,m)\in {𝒯}_{G}}log\left(1-{𝒟}_{\psi }(s,a,m)\right)-\beta H\left(\pi_{\theta }\right)$$

where ${𝒯}_{E}$ and ${𝒯}_{G}$ are the observed true trajectories and the trajectories generated by the policy network $\pi_{\theta }$ under the moving behavior m, respectively. $H\left(\pi_{\theta }\right)$ is the entropy regularization term, which controls finding the policy with maximum causal entropy.

### 2.1 Policy Network

#### 2.1.1 Mobility Trajectory Encoder.

The mobility trajectory encoder encodes historical mobility movements and generates a density vector representing the likelihood for the next location. At each timestamp, the mobility trajectory encoder first embeds the location ID $l_{i}$, time $t_{i}$, and moving behavior m via embedding layers and concatenates them into a dense representation. We then apply RNN networks to predict sequential actions with spatiotemporal context [19]. Lastly, a multilayer perceptron (MLP) with a softmax function is used to transform the latent vector $h_{i}^{L}$ into a density vector $\lambda_{i}^{L}\in R^{K}$ that represents the likelihood of going to location $l_{i}$ at time $t_{i}$ that is learned from the mobility trajectory.

#### 2.1.2 Context Predictor.

As human mobility reveals the functions and properties of urban regions [15], we consider the context of a location to be an important factor underlying the decision process. For example, a work commute trajectory generally travels and stays in the work area and residential area, resulting in locations with those designated region functionality having a higher likelihood to appear in the trajectory. To model the simulation process guided by contextual information, we train a context predictor that shares a similar model to the RNN followed by MLP architecture used in the mobility trajectory encoder. The context predictor takes the past context trajectory $\tau^{C}$ and the moving behavior m as input and generates a probability vector $p^{C}$ that predicts the likelihood of the next context type. Then this context-type probability vector is mapped to a density vector $\lambda^{C}$ to guide the location prediction in the next step.

#### 2.1.3 Spatial Dynamics Enforcer.

We also consider spatial continuity as an important factor in generating realistic trajectories. For example, the trajectory of movements is usually limited by a speed threshold; i.e., traveling between two consecutive points in the trajectory must be physically feasible for the moving object. To learn the stochastic constraints, MBP-GAIL generates a density vector $\lambda^{S}$ at each step where each element of the vector follows a parameterized Gaussian distribution $𝒩\left(0,\sigma_{i}^{S}\right)$ with a moving distance. The Gaussian distribution is centered at zero, where closer proximity of the next location to the current ones gives a higher value in the spatial density vector of the corresponding location and, consequently, indicates the higher likelihood of this location grid being chosen in the next step.

#### 2.1.4 Density Fusion.

Density fusion is the final part of the policy network that combines the three density vectors obtained from the previous steps. It fuses the three vectors by multiplying them all together to generate a weighted density $\lambda_{i}\in R^{K}\lambda_{i}=\lambda_{i}^{L}⊙\lambda_{i}^{C}⊙\lambda_{i}^{S}$ and get the probability vector for sampling a at step i as $p_{i}=softmax\left(\mu \lambda_{i}\right)$ where μ is a scaling factor.

### 2.2 Discriminator and Moving Behavior Classifier

GAIL uses a reward function to evaluate the actions by comparing the policy-generated actions with real-world actions. It is first modeled by a discriminator 𝒟, which aims to distinguish between real and generated samples. The input of the discriminator is the state action tuple (s, a) of both real-world and policy-generated trajectories. Like the policy network, we leverage an RNN to encode the state history and replace the MLP layer with a binary classifier. Following [1], we utilize a sigmoid cross entropy to sample positive samples from observed trajectories and negative samples from generated trajectories. Optimization is carried out with the following loss function with gradient descent, where we define $r_{D}=-log\left(1-{𝒟}_{\psi }(s,a,m)\right)$

#### Moving behavior Classifier

Since the current discriminator 𝒟 focuses only on estimating how realistic a generated sequence is, it may lose some of the original moving behavior pattern preserved in context sequences while generating trajectories. To preserve the moving behavior content, we leverage a multi-class classifier 𝒞, which has a structure similar to the discriminator 𝒟 and trains on the context sequences converted from real-world trajectories with their associated moving behavior labels. Here for 𝒞, unlike the discriminator, which uses a binary cross-entropy loss, we perform the prediction of moving behavior by using softmax cross-entropy loss and incorporate the reward term of moving behavior into the learning of our policy as follows $r_{C}=argmax\left(C\left(\tau^{L}\right)⊙OneHot(m)\right)$ where $C\left(\tau^{L}\right)$ is the output of the classifier which is an M dimensional vector encoding the classified probability distribution.

The generator will be rewarded with such a design if the generated trajectory satisfies the moving behavior pattern property. The final reward for training the policy net $\pi_{\theta }$ is defined as follows: $r=(1-v)\;*\;r_{C}+v\;*\;r_{D}$ where v helps balance the objective of satisfying the constraints of moving behaviors and mimicking the true trajectories, both of which push the policy learning towards modeling more realistic transitions.

## 3 EXPERIMENTS

### 3.1 Experimental Setting

#### Dataset.

We collect mobility trajectories in Houston and Los Angeles from Veraset^1^ in March 2020. We uniformly sample 15,000 trajectories due to the large data size. We divide the study region into equal side-length (spatial) grid cells with a given side-length l=200 meters and discretize the time cycle of a trajectory into periods of one minute for 60 intervals (one hour) to represent temporal information. POI information of the located regions can be accessed from the Safegraph open website. ^2^.

#### Baselines.

We compare MBP-GAIL with the following baseline methods: **Markov Model** It defines all visited locations as states and builds a transition matrix to capture the transition probabilities between them. **LSTM** A widely used sequential neural network that predicts the next location given historically visited locations **TransVAE** [13, 16] A variational autoencoder (VAE)-based generative model where the encoder and decoder are designed with the Transformer architecture. **SeqGAN** [17] A sequence generative adversarial network to generate the next location based on past states **MoveSim** [1] A GAN-based generator that incorporates domain knowledge, such as the urban structure of the regions and POI information in the model.

### 3.2 Performance Comparison

#### 3.2.1 Evaluation Metric.

Our goal is to generate activity trajectories similar to real-world activities. We adopt these evaluation metrics to evaluate the quality of generated data: **Distance:** the cumulative travel distance per trajectory. **Radius:** radius of gyration, which measures the spatial range. **Duration:** stay duration, which is calculated as the duration of stay per visit to the location. P(r): the visiting probability of one location $r.\;P\left(r_{1},r_{2}\right)$: the probability that a trajectory transitions from location $r_{1}$ to location $r_{2}$

We use the Jensen–Shannon divergence (JSD) to measure the similarity between the mobility pattern distributions of the generated trajectory and the real-world trajectory data, which is defined as $JSD(p∥q)=H((p+q)/2)-\frac{1}{2}(H(p)+H(q))$ where H is Shannon information, p and q are distributions. Lower JSD indicates a better generation result.

#### 3.2.2 Evaluation Results.

In this section, we investigate the performance of MBP-GAIL on dataset-level evaluation of real-world data. Table 1 shows the performance of MBP-GAIL, traditional baseline methods. As we can observe from Table 1, the Markov Model performs the worst across all metrics, indicating that simply conditioning on one previous location cannot generate meaningful and realistic trajectories. Movesim achieves second-best performance on most of these metrics and consistently performs better than SeqGAN and TrajGAIL, which validates the importance and necessity of incorporating domain knowledge, such as spatial continuity and temporal periodicity, in the generation process. Despite this, MBP-GAIL achieves consistent performance improvements over state-of-the-art prediction and generation methods, especially in Houston. For example, MBP-GAIL significantly improves the JSD metrics evaluation for the distance over the best baseline, Movesim, by 69%

## 4 CONCLUSION AND LIMITATIONS

This paper presented a novel generative adversarial framework dubbed as MBP-GAIL, designed to synthetize human mobility trajectories. MBP-GAIL captures the underlying patterns of movement behavior, a crucial aspect in generating realistic and representative mobility data. We emphasize the importance of integrating moving behavior and spatial constraints in generating massive amounts of mobility data that closely resemble real-world scenarios. Through extensive experiments, we have demonstrated the exceptional performance of MBP-GAIL in generating synthetic mobility data.

## Figures and Tables

**Table 1:** Performance comparison on two mobility datasets. Bold denotes the best (lowest). The underscore denotes the second-best.

	Houston					Los Angeles				

	Distance	Radius	Duration	*P* (*r*)	*P* (*r*_1_, *r*_2_)	Distance	Radius	Duration	*P* (*r*)	*P* (*r*_1_, *r*_2_)

Markov Model	0.5098	0.5032	0.4428	0.0028	0.3280	0.4086	0.4122	0.4332	0.0046	0.3073
LSTM	0.4865	0.4050	0.3748	**0.0023**	0.0881	0.3855	0.3050	0.3830	0.0032	0.1044
TransVAE	0.4662	0.3942	0.3276	0.0034	0.1537	0.3872	0.3443	0.3539	0.0042	0.1462
SeqGAN	0.3318	0.2908	0.2160	0.0074	0.1055	0.2948	0.1913	0.1490	0.0025	0.0910
MoveSim	0.2413	0.2402	0.1520	0.0025	0.0924	0.0922	**0.1274**	0.1617	**0.0021**	0.0932
MBP-GAIL	**0.0744**	**0.1215**	**0.1311**	0.0024	**0.0874**	**0.0667**	0.1305	**0.1452**	0.0023	**0.0891**

## CCS CONCEPTS

**• Information systems → Geographic information systems; • Applied computing →** *Trajectory generation.*

## Footnotes

1
https://www.veraset.com/about-veraset

2
https://docs.safegraph.com/v4.0/docs/places-schema-section-patterns
